# Left atrioventricular interaction and impaired left atrial phasic function in type 2 diabetes mellitus patients with or without anemia: a cardiac magnetic resonance study

**DOI:** 10.1186/s12933-023-01910-8

**Published:** 2023-07-13

**Authors:** Wen-Lei Qian, Zhi-Gang Yang, Rui Shi, Ying-Kun Guo, Han Fang, Meng-ting Shen, Yuan Li

**Affiliations:** 1 Department of Radiology, West China Hospital, Sichuan University, 37# Guo Xue Xiang, Chengdu, Sichuan 610041 China; 2 Department of Radiology, Key Laboratory of Obstetric & Gynecologic and Pediatric Diseases and Birth Defects of Ministry of Education, West China Second University Hospital, Sichuan University, 20# South Renmin Road, Chengdu, Sichuan 610041 China

**Keywords:** Type 2 diabetes mellitus, Anemia, Atrioventricular interaction, Left atrial phasic function, Cardiac magnetic resonance

## Abstract

**Objective:**

Type 2 diabetes mellitus (T2DM) and anemia are related to some cardiovascular diseases and can predict poor outcomes. Both of them can damage the heart in their own ways, but their combined effects have not been well explored. This study aimed to explore the combined effects of T2DM and anemia and the interaction between left atrial (LA) and left ventricular (LV) function by cardiac magnetic resonance (CMR).

**Materials and methods:**

A total of 177 T2DM patients without anemia, 68 T2DM patients with anemia and 73 sex-matched controls were retrospectively enrolled in this study from June 2015 to September 2022. Their LA phasic function and LV function parameters were compared to explore the combined effects of T2DM and anemia and the interaction between LA and LV function. Univariate and multivariate linear regression were done to explore the independent factors influencing LA phasic function and LV function.

**Results:**

Compared with controls and T2DM patients without anemia, T2DM patients with anemia were older and had higher heart rate, higher creatinine, lower estimated glomerular filtration rate (eGFR) and lower hemoglobin (Hb) (all p < 0.05). LV global longitudinal peak strain (GLPS) significantly declined from T2DM patients with anemia to T2DM patients without anemia to controls (p < 0.001). LA volumetric function and strain were significantly impaired in T2DM patients with anemia compared with the other groups (all p < 0.05). In addition to age, eGFR, Hb and HbA1c, the LV GLPS was independently associated with all LA phasic strains (LA reservoir strain, β =0.465; LA conduit strain, β = 0.450; LA pump strain, β = 0.360, all p < 0.05). LA global conduit strain, total LA ejection fraction (LAEF) and active LAEF were independently associated with LV GLPS and LVEF.

**Conclusion:**

Both LA and LV function were severely impaired in T2DM patients with anemia, and T2DM and anemia were independently associated with LA phasic function. Deleterious interaction between LA function and LV function would happen in T2DM patients with or without anemia. Timely and effective monitoring and management of both LA and LV function will benefit T2DM patients.

**Supplementary Information:**

The online version contains supplementary material available at 10.1186/s12933-023-01910-8.

## Introduction

Type 2 diabetes mellitus (T2DM) is a worldwide healthcare burden due to its high prevalence, high mortality and high morbidity [1, 2]. Established cardiovascular disease happened in about one third of T2DM patients [3]. Nearly half of the deaths of patients with T2DM are related to cardiovascular disease [4, 5]. Anemia is also a worrying clinical condition. According to one study, approximately 27% of the world’s population (1.93 billion people) suffered from anemia in 2013 [6]. It can lead to nonhemodynamic and hemodynamic changes to compensate for the insufficient oxygen supply [7]. It is associated with increased all-cause mortality in the general population [8] and can predict adverse cardiovascular outcomes [9]. When combined with T2DM, the pooled prevalence of anemia is as high as 33% [10], and the cardiovascular system may suffer double damage from T2DM and anemia [11].

The left atrium (LA) plays an important role in the cardiac cycle. It not only has endocrine functions (atrial natriuretic peptide synthesis and secretion) and regulatory functions (such as regulation of autonomic nervous system activity and reflex control of the circulation), but can mechanically regulate left ventricular (LV) filling and cardiac output [12]. LA size and function have been recognized as significant prognostic markers in many cardiovascular diseases [13, 14]. Furthermore, LV function can in turn influence LA function because LA phasic function depends heavily on LV performance [15]. Thus, any atrioventricular coupling problem would damage both LA and LV function and even form a vicious circle.

Many studies have focused only on the damage to the heart caused by T2DM or anemia [7, 16], while few studies have explored the combined effects of anemia and T2DM on the heart [11]. Among studies of left heart function, Many studies have also focused only on the left ventricle or left atrium, leaving the interaction of the LV with the LA poorly explored, especially by cardiac magnetic resonance (CMR). Moreover, to the best of our knowledge, no study has explored the combined effects of T2DM and anemia on left atrioventricular interaction. Thus, this study aimed to [1] explore the combined effects of T2DM and anemia on the left heart and [2] explore the left atrioventricular interaction in T2DM patients with or without anemia by CMR feature tracking technology.

## Materials and methods

### Study Population

The West China Hospital of Sichuan University biomedical research ethics committee approved this study protocol. Informed consent was waived due to the retrospective nature of the research.

The inclusion and exclusion criteria of all our participants were similar to those of a previous study [17]. Specifically, the inclusion criteria for all T2DM patients were as follows: [1] patients met the diagnostic criteria of Standards of Medical Care in Diabetes [18], [2] underwent CMR examination, and [3] had complete medical records. The exclusion criteria for all T2DM patients were [1] history of congenital heart diseases, primary myocardiopathy or secondary myocardiopathy not caused by T2DM, severe aortic or mitral valve diseases, or severe renal failure (estimated glomerular filtration rate (eGFR) < 30 ml/min), [2] incomplete clinical records; and [3] contraindications to CMR or poor CMR image quality. The inclusion criteria for the control group were as follows: [1] no T2DM history or impaired fasting glucose; [2] no history of diseases that could impair cardiac function, such as coronary heart disease, hypertension, valvular heart disease, cardiomyopathy, metabolic disease, and systemic diseases; and [3] normal cardiac function. Anemia was diagnosed by the WHO criteria [hemoglobin (Hb) concentration less than 120 g/l in nonpregnant adult females and 130 g/l in adult males] [19]. Finally, a total of 177 T2DM patients without anemia (84 females, 47.5%), 68 T2DM patients with anemia (31 females, 45.6%) and 73 sex-matched controls (37 females, 50.7%) were consecutively enrolled in this investigation from June 2015 to September 2022.

### CMR Protocol

All CMR examinations were performed by either of two 3.0-T whole-body scanners (MAGNETOM Skyra and MAGNETOM Trio Tim; Siemens Medical Solutions, Erlangen, Germany) with a 32-channel body phased-array coil in the supine position. To get high-quality CMR images, standard electrocardiogram-triggering devices were used to monitor the participants’ electrocardiograms, and the data were collected during breath-hold intervals. CMR cine images were obtained from a steady-state free precession (SSFP) sequence. The parameters were as follows: temporal time = 39.34/40.35 ms; echo time = 1.22/1.20 ms; slice thickness = 8.0 mm; field of view = 234 × 280/250 × 300 mm2; matrix size = 208 × 139/192 × 162 pixels; and flip angle = 39°/50°. A stack of parallel slices including LV two-chamber short-axis views and four-chamber, three-chamber and two-chamber long-axis views were obtained from the cine images.

### CMR Analysis

#### LA and LV volumetric function analysis

Commercial software (cvi42, v.5.11.2; Circle Cardiovascular Imaging, Inc., Calgary, AB, Canada) was used for all CMR analyses. The biplane area-length method was used to automatically calculate the LA volumes of three phases [20] in the cvi42 biplanar LAX module. All LA phasic volumes were indexed to body surface area (BSA). The maximum LA volume (LAV_max_) was measured at LV end-systole, the pre-atrial contraction LA volume (LAV_pre−a_) was measured before the initiation of atrial contraction, and the minimum LA volume (LAV_min_) was measured at LV end-diastole. The LA appendage and pulmonary veins were excluded from the LA volumes. The LA emptying fractions were then calculated as in the followings formulas [21]:

1. total LA emptying fraction (total LAEF) = (LAV_max_ - LAV_min_)/LAV_max_*100%;
2. passive LA emptying fraction (passive LAEF) = (LAV_max_ -LAV_pre-a_)/LAV_max_*100%;
3. active LA emptying fraction (active LAEF) = (LAV_pre-a_ - LAV_min_)/LAV_pre-a_*100%.

Total LAEF corresponds to atrial reservoir function, passive LAEF corresponds to atrial conduit function, and active LAEF corresponds to atrial contractile pump function.

The endocardium and epicardium at LV end-systole and LV end-diastole on the two-chamber short axis were automatically delineated and manually corrected to calculate LV volumetric function parameters, including LV end-diastolic volume (LVEDV), LV end-systolic volume (LVESV), LV stroke volume index (LVSV), LV ejection fraction (LVEF), LV mass (LVM), cardiac output (CO) and cardiac index (CI). LVEDV, LVESV, LVSV and LVM were indexed to BSA (corresponding to LVEDVI, LVESVI, LVSVI and LVMI, respectively).

#### LA and LV feature tracking analysis

**Fig. 1:**
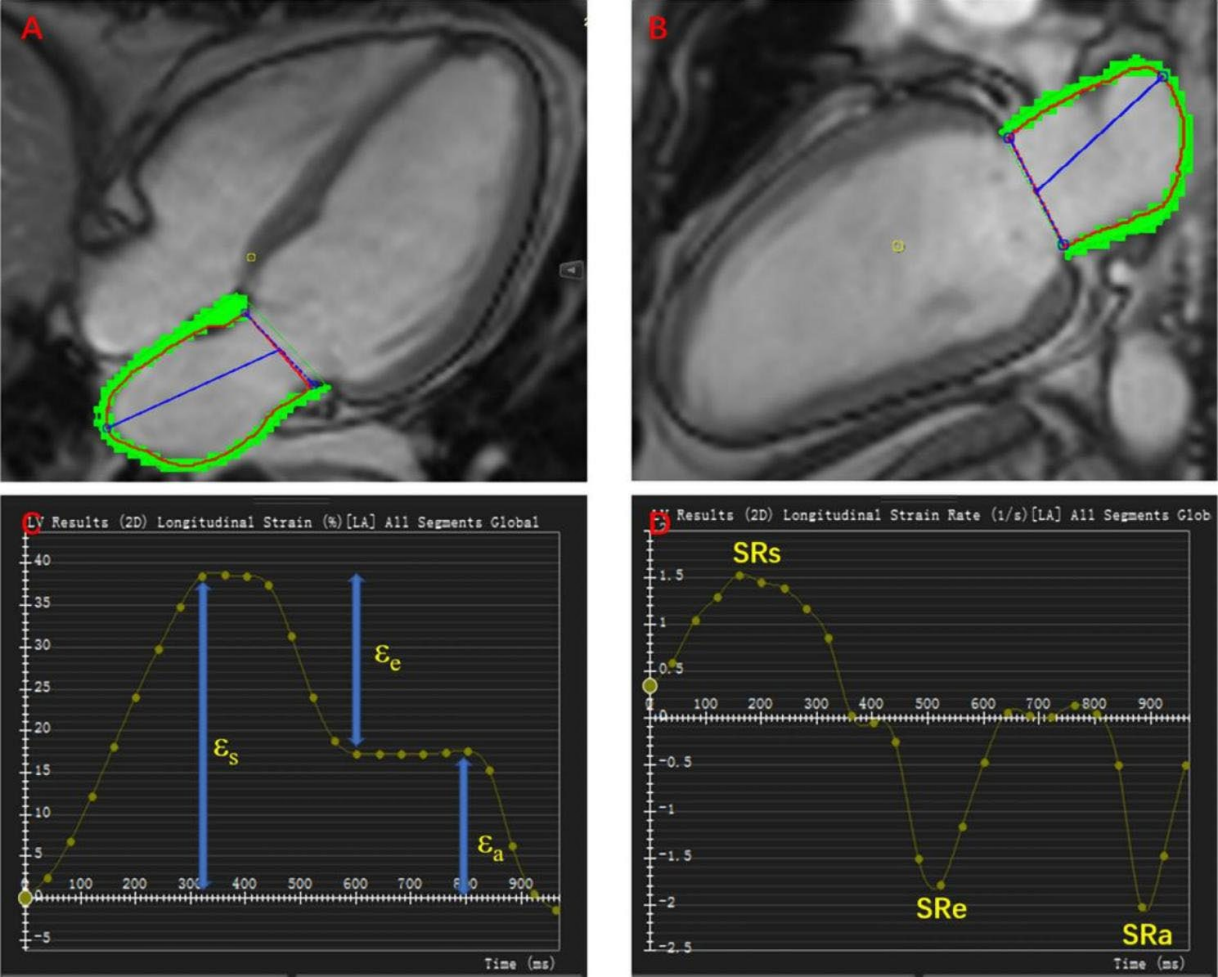
Example pictures of LA strain and strain rate in a T2DM patient without anemia. A and B are two CMR cine pseudo-colour images in four-chamber long axis and two-chamber long axis respectively; C is the strain-time curve and D is the strain rate-curve of LA. LA, left atrial; T2DM, type 2 diabetes mellitus; CMR, cardiac magnetic resonance

The tissue tracking module of cvi42 was used to analyze LA and LV myocardial strains. The epicardial and endocardial borders of the LA were manually delineated by a point-and-click approach in apical two-chamber and four-chamber views (Fig. 1). The LA appendage and pulmonary veins were also excluded. Then, an automated tracking algorithm was applied to delineate the atrial borders in the subsequent slices. To ensure these strain parameters were accurate, an experienced cardiac MR radiologist (QWL) reviewed the tracking performance to ensure the accuracy of automated tracking and manually adjusted any inaccurate tracking. The LA global longitudinal strain of three phases were used for analyses, including LA reservoir strain (ε_s_) (corresponding to LA reservoir function), passive strain (ε_e_) (corresponding to LA conduit function), and active strain (ε_a_) (corresponding to LA pump function).

At LV end-diastole, the endocardium and epicardium of the two-chamber short-axis, two-chamber long-axis and four-chamber long-axis cine slices were automatically drawn and manually corrected to calculate the LV global longitudinal peak strain (GLPS).

### Reproducibility

Two experienced radiologists (QWL and SR) who had at least 3 years of CMR experience and were blinded to the patients’ clinical data carefully delineated the epicardial and endocardial borders of the LA of 45 randomly selected participants. The CMR images of 10 controls, 10 T2DM patients with anemia and 25 T2DM patients without anemia were used to assess the intraobserver and interobserver variabilities. QWL delineated the 45 participants’ CMR images twice 1 month apart to assess the intraobserver variability. The interobserver variability was assessed by comparing the data from SR (who was blinded to the results of QWL) and QWL.

### Statistical analysis

The distribution of continuous data was tested by the Shapiro‒Wilk test. Normally distributed continuous data are expressed as the mean ± standard deviation, and nonnormally distributed continuous data are presented as the median (25 – 75% interquartile range). The independent T test (normally distributed data) and the Mann‒Whitney U test (nonnormally distributed data) were used to compare continuous data of the two T2DM groups. To compare normally distributed continuous data between the controls, T2DM patients without anemia and T2DM patients with anemia, one-way analysis of variance (ANOVA) with Bonferroni’s (homogeneity of variances) or Tamhane’s T2 post hoc correction (heterogeneity of variances) was used. To compare nonnormally distributed data between the three groups, the Kruskal‒Wallis test was used. Categorical variables are expressed as frequencies (percentages) and were analyzed using the chi-square test. Pearson’s and Spearman’s correlation coefficients, according to the distributions of data, were calculated between LA global longitudinal strains (ε_s_, ε_e_, and ε_a_), clinical indices (such as sex, age, plasma lipid parameters, Hb, HbA1c and so on), and LV global longitudinal strain parameters (GLPS). To identify the independent predictors of LA strains and LV functional parameters, data were input into multivariate linear regression analyses using stepwise selection or the enter method if p < 0.1 in univariate linear regression. Intraclass correlation coefficients (ICCs) were calculated to measure the interobserver and intraobserver agreements. SPSS version 25 (IBM, Armonk, New York, USA) was used to perform all analyses, and a two-tailed p < 0.05 was considered indicative of significance. Prism software (version 9.0.0 (121), GraphPad Software Inc., San Diego, CA, USA) was used to draw the scatter plot of the correlation between LV and LA function and the comparison of LA global strain parameters (ε_s_, ε_e_, and ε_a_) between the three subgroups.

## Results

### Baseline clinical characteristics

Between the three groups, age, heart rate, systolic blood pressure (SBP), diastolic blood pressure (DBP), plasma lipid parameters, estimated glomerular filtration rate (eGFR), creatinine and Hb were significantly different (all p < 0.05; the details are shown in Table 1). While sex, body mass index (BMI) and smoking history showed no significant difference. T2DM patients with anemia wereolder and had a higher heart rate, higher creatinine, lower eGFR and lower Hb than the controls and T2DM patients without anemia (Hb: 105 ± 13 g/L vs. 140 ± 14 g/L vs. 139 ± 12 g/L, p < 0.001), while the latter two groups were similar in all these values. When comparing the two groups with T2DM, diabetes duration, HbA1c and medications showed no significant difference (all p > 0.05).

**Table 1:** Baseline characteristics of the study population

	Controls (n = 73)	T2DM Without Anemia (n = 177)	T2DM With Anemia (n = 68)	P value
Demographics				
Female, n(%)	37(50.7)	84(47.5)	31 (45.6)	0.825
Age, years	55.95 ± 9.02	57.29 ± 10.58	63.47 ± 11.82*^§^	0.000
BMI (kg/m2)	23.41 ± 3.06	24.54 ± 2.95*	24.23 ± 3.34	0.038
Heart rate (beats/min)	72 ± 14	74 ± 14	79 ± 15*^§^	0.008
SBP (mmHg)	119 ± 13	130 ± 18*	127 ± 18*	<0.001
DBP (mmHg)	75 (69–81)	78 (71–86)	74 (65–82) ^§^	0.017
Smoking history, n (%)	14 (19.2)	47 (26.6)	22(32.4)	0.201
Diabetes duration, years	–	6 (2.0–10.0)	5.5 (2.0–9.0)	0.192
Laboratory data				
Hb, (g/L)	140 ± 14	139 ± 12	105 ± 13*^§^	<0.001
HbA1c, %	–	7.45 ± 1.63	6.99 ± 1.17	0.126
TG (mmol/L)	1.39 (1.01– 1.75)	1.53 (1.00–2.24)	1.22 (0.92–1.74)	0.067
TC (mmol/L)	4.87 (4.03–5.35)	4.19 (3.46–5.01) *	3.55 (3.09–4.48) *^§^	<0.001
HDL (mmol/L)	1.27 (1.11–1.56)	1.16 (0.93–1.42) *	1.13 (0.89–1.40) *	0.002
LDL (mmol/L)	2.94 (2.29–3.43)	2.33 (1.72–2.95) *	1.86 (1.46–2.32) *^§^	<0.001
eGFR (mL/min/1.73 m^2^)	96.5 (83.0–105.6)	91.8 (72.3–102.8)	67.1 (51.3–88.9) *^§^	<0.001
Creatinine, (umol/L)	70.0 (58.5–80.0)	73.0 (60.0–88.0)	98.0 (69.0–114.0) *^§^	<0.001
Medications, n (%)				0.401
Insulin	–	30 (16.95)	14 (25.00)	
Biguanides	–	72 (40.68)	28 (41.18)	
Sulfonylureas	–	31 (17.51)	6 (8.82)	
α‑Glucosidase inhibitor	–	33 (18.64)	11(16.18)	
Others	–	27 (15.25)	9(13.24)	
No	–	33 (18.64)	19 (27.94)	

Note: Data are presented as the mean ± SD, median (Q1 – Q3) or number (percentage)

*P less than 0.017 vs. controls; ^§^P less than 0.017 vs. T2DM patients without anemia

BMI, body mass index; SBP, systolic blood pressure; DBP, diastolic blood pressure; Hb, hemoglobin; TC, total cholesterol;

TG, triglyceride; HDL, high-density lipoprotein; LDL, low-density lipoprotein cholesterol; eGFR, estimate glomerular filtration rate;

### Comparisons of CMR-derived LA and LV volumetric function between the three groups

**Fig. 2:**
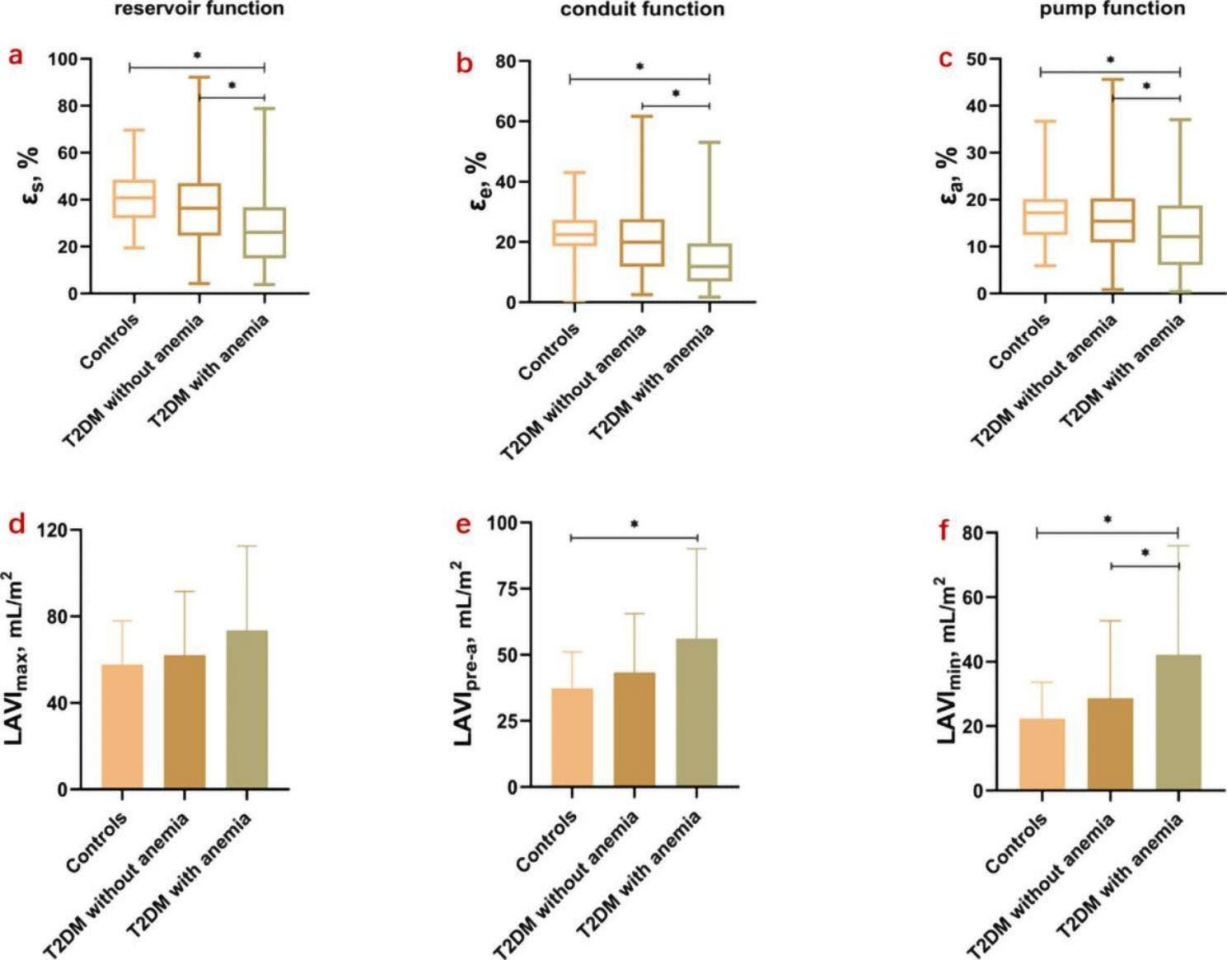
Comparison of reservoir function, conduit function and pump function among controls, T2DM patients without anemia and T2DM patients with anemia. a – c, LA strain in three phases, d – f, LAVI in three phases. a and d represent LA reservoir function, b and e represent LA conduit function, c and f represent LA pump function. ε_s_, total strain; ε_e_, passive strain; ε_a_, active strain; LAV, left atrial volume; I, index to body surface area. * means P less than 0.05

For LA volumetric function, the LAVI_min_ was significantly increased, while the total LAEF, passive LAEF and active LAEF were significantly decreased in T2DM patients with anemia, compared with T2DM patients without anemia and controls (all p < 0.05). LAVI_pre−a_ was higher in T2DM patients with anemia than controls, while T2DM patients without anemia were not significantly different from controls or T2DM patients with anemia [38.0 (27.1–47.1) mL/m^2^ vs. 39.5 (28.1–51.2) mL/m^2^ vs. 46.6 (32.8–91.0) mL/m^2^, p = 0.006]. There was no significant difference between the three groups in LAV_max_ (p = 0.113, Fig. 2).

For LV volumetric function, the LVEDVI and CO of T2DM patients with anemia were significantly higher than those of T2DM patients without anemia but were similar to those of controls (p = 0.030 and 0.001, respectively). The LVSVI and CI of T2DM patients with anemia were also similar to that of controls, while the T2DM patients without anemia had the lowest LVSVI and CI (all p < 0.001). LVESVI was higher in T2DM patients without anemia than controls and T2DM patients with anemia, while the latter two groups showed no significant difference (p = 0.033). The two T2DM groups had a lower LVEF than the control group, and they all had mean LVEF higher than 50% (61.9 ± 7.1% vs. 54.0 ± 12.4 vs. 52.9 ± 12.4%, p < 0.001, Table 2).

**Table 2:** Comparisons of CMR derived LA and LV function between three groups

	Controls (n = 73)	T2DM Without Anemia (n = 177)	T2DM With Anemia (n = 68)	P value
LV volumetric function				
LVEDVI, mL	82.7 (70.7–91.4)	77.1 (65.6–94.4)	89.1 (69.1–112.7) ^§^	0.030
LVESVI, mL	30.7 (25.0–37.4)	32.2 (25.1–42.8)	35.8 (26.9–57.4) *	0.033
LVSVI, mL	50.5 ± 9.6	44.1 ± 10.5 *	48.3 ± 14.3	< 0.001
CO, L/min	5.9 ± 1.5	5.5 ± 1.3	6.3 ± 1.8 ^§^	0.001
CI, L/(min*m^2^)	3.6 ± 0.9	3.2 ± 0.8 *^§^	3.7 ± 1.1	< 0.001
LVEF, %	61.9 ± 7.1	54.0 ± 12.4 *	52.9 ± 12.4 *	< 0.001
LVMI, g	43.1 (38.8–49.3)	48.4 (38.1–59.8) *	53.3 (45.3–64.6) *^§^	< 0.001
LV strain parameters LV GLPS (%)	-13.15 ± 2.50	-10.87 ± 4.76 *	-9.14 ± 3.97 *^§^	< 0.001
LA phasic volume, mL/m^2^				
LAVI_max_	55.7 (43.0–71.1)	57.1 (42.1–73.1)	63.3 (46.3–89.2)	0.113
LAVI_pre−a_	38.0 (27.1–47.1)	39.5 (28.1–51.2)	46.6 (32.8–91.0) *	0.006
LAVI_min_	20.6 (15.0–27.6)	23.7 (14.7–31.9)	31.4 (15.8–58.4) *^§^	0.001
LAEF, %				
Total LAEF	63.5 (58.5–67.1)	59.6 (52.0–67.2)	49.4 (36.2–61.5) *^§^	< 0.001
Passive LAEF	35.0 ± 9.6	32.0 ± 16.2	25.4 ± 11.5 *^§^	< 0.001
Active, LAEF	43.5 (33.7–50.4)	43.6 (35.8–51.0)	32.2 (15.8–43.7) *^§^	< 0.001
LA global longitudinal strain, %				
ε_s_	41.06 ± 12.17	36.84 ± 17.33	27.16 ± 16.53 *^§^	< 0.001
ε_e_	22.40 (18.53–27.35)	19.90 (11.70–27.60)	11.85 (6.83–19.58) *^§^	< 0.001
ε_a_	17.20 (12.20–20.20)	15.40 (10.80–20.30)	12.10 (6.03–18.80) *^§^	0.006

Note: Data are presented as the mean ± SD, or median (Q1 – Q3)

*P less than 0.017 vs. controls; ^§^P less than 0.017 vs. T2DM patients without anemia

T2DM, type 2 diabetes mellitus; LV, left ventricular; EDV, end diastolic volume; ESV, end systolic volume; SV, stroke volume; CO, cardiac output; CI, cardiac index; EF, ejection fraction; M, mass; LAV, left atrial volume; I, indexed to body surface area; LAEF, left atrial emptying fraction; GLPS, global longitudinal peak strain; ε_s_, total strain; ε_e_, passive strain; ε_a_, active strain

### Comparisons of CMR-derived LA and LV global longitudinal strains between the three groups

Regarding LA global longitudinal strain, T2DM patients with anemia had lower ε_s_ (27.16 ± 16.53% vs. 36.84 ± 17.33% vs. 41.06 ± 12.17%), ε_e_ [11.85 (6.83–19.58)% vs. 19.90 (11.70–27.60)% vs. 22.40 (18.53–27.35)%] and ε_a_ [12.10 (6.03–18.80)% vs. 15.40 (10.80–20.30)% vs. 17.20 (12.20–20.20)%] than T2DM patients without anemia and controls, while the latter two groups showed no significant difference (all p < 0.05, Fig. 2). As for parameters of LV global longitudinal strain, the GLPS (-13.15 ± 2.50% vs. -10.87 ± 4.76% vs. -9.14 ± 3.97%) was significantly decreased from controls to T2DM patients without anemia to T2DM patients with anemia (p < 0.001). The details are shown in Table 2.

### Association between LA strains and other variables in all T2DM patients

**Fig. 3:**
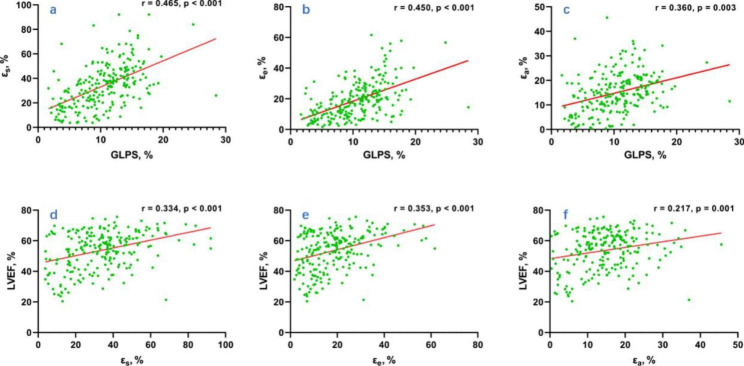
Interaction between LA function and LV function. a – c, linear regression analysis between the magnitude of LV GLPS and LA phasic strain; d – f, linear regression analysis between the magnitude of LA phasic strain and LVEF. LA, left atrial; LV, left ventricular; GLPS, global longitudinal peak strain, ε_s_, total strain; ε_e_, passive strain; ε_a_, active strain

By univariate and multivariate linear regression analyses, age, DBP, HbA1c, eGFR, Hb and LV GLPS were independently associated with LA ε_s_ and ε_e_ (all p < 0.05). Age, DBP, Hb and LV GLPS were independently associated with LA ε_a_ (all p < 0.05). LV GLPS had the greatest influence on ε_s_, ε_e_ and ε_a_ (β = 0.465, 0.450 and 0.360, respectively, all p < 0.05, Table 3; Fig. 3).

**Table 3:** Univariate and multivariate linear regression analyses of LA global longitudinal strain in all T2DM patients

	ε_s_				ε_e_				ε_a_				
	**Univariable**		**Multivariable**		**Univariable**		**Multivariable**			**Univariable**		**Multivariable**	
	**β**	P value	**β**	P value	**β**	P value	**β**	P value		**β**	P value	**β**	P value
Sex	0.111	0.091			0.158	0.005*	0.085	0.234		0.002	0.973		
Age	0.247	< 0.001*	0.177	0.005*	0.233	< 0.001*	0.167	0.008^*^		0.195	0.003*	0.176	0.019*
BMI	0.017	0.796			0.073	0.271				-0.057	0.390		
Heart rate	0.006	0.926			-0.009	0.893				0.014	0.838		
SBP	0.023	0.733			0.027	0.694				0.021	0.762		
DBP	0.211	0.002*	0.157	0.013*	0.204	0.002*	0.131	0.033		0.164	0.017*	0.155	0.039*
Diabetes duration	0.062	0.406			0.035	0.635				0.107	0.152		
HbA1c	-0.208	0.003*	-0.233	< 0.001*	-0.214	0.002*	-0.259	< 0.001*		-0.142	0.047*	-0.137	0.070
Hb	0.176	0.010*	0.173	0.018*	0.180	0.009*	0.207	0.009*		0.149	0.032*	0.179	0.042*
eGFR	0.357	< 0.001*	0.173	0.015*	0.406	< 0.001*	0.225	0.001*		0.195	0.004*	0.041	0.625
TG	-0.049	0.462			-0.054	0.411				-0.011	0.868		
TC	0.247	< 0.001*	0.063	0.570	0.265	< 0.001*	-0.009	0.931		0.159	0.017*	0.133	0.306
HDL	0.257	< 0.001*	-0.021	0.852	0.277	< 0.001*	0.014	0.842		0.168	0.011*	-0.047	0.579
LDL	0.224	< 0.001*	-0.057	0.605	0.226	< 0.001*	-0.024	0.821		0.156	0.019*	-0.117	0.370
LVEDVI	-0.265	< 0.001*			-0.238	< 0.001*				-0.258	< 0.001*		
LVESVI	-0.354	< 0.001*	-0.046	0.683	-0.348	< 0.001*	0.030	0.785		-0.294	< 0.001*	-0.167	0.213
LVSVI	0.042	0.537			0.107	0.112				0.008	0.910		
LVMI	-0.348	< 0.001*	-0.041	0.614	-0.335	< 0.001*	-0.025	0.765		-0.269	< 0.001*	0.012	0.900
LVEF	0.358	< 0.001*	-0.088	0.417	0.354	< 0.001*	-0.033	0.279		0.218	0.001*	-0.174	0.174
CI	0.025	0.706			0.070	0.297				-0.027	0.693		
CO	-0.034	0.618			0.007	0.913				-0.060	0.379		
LV GLPS	0.563	< 0.001*	0.465	< 0.001*	0.559	< 0.001*	0.450	< 0.001*		0.407	< 0.001*	0.360	0.003*

Note: Abbreviation of BMI, SBP, DBP, Hg, eGFR, TG, TC, HDL, LDL are shown in Table 1; LVEDVI, LVESVI, LVSVL, LVMI, LVEF, CI, CO, GLPS, ε_s_, ε_e_, and ε_a_ are showing in Table 2

* P less than 0.05

### Association between LV function and LA phasic function in all T2DM patients

All LA phasic function parameters were included in the multivariate linear regression model using stepwise collection (Table 4). LA function parameters were independently associated with LV function to varying degrees. Among them, ε_e_ (β = 0.109, p = 0.001) and total LAEF (β = 7.040, p = 0.003) were independently associated with LV GLPS, with a model’s coefficient of determination (R^2^) of 0.215. ε_e_ (β = 0.277, p < 0.001) and active LAEF (β = 12.599, p = 0.023) were independently associated with LVEF, R^2^ = 0.134.

**Table 4:** Associations between LV function and LA phasic function in all T2DM patients

	GLPS			LVEF	
	β (95% CI)	R^2^		β (95% CI)	R^2^
**Model 1**		0.187	**Model 1**		0.117
ε_e_	0.171 (0.124–0.217) *		ε_e_	0.361 (0.230–0.492) *	
**Model 2**		0.215	**Model 2**		0.134
ε_e_	0.109 (0.048–0.170) *		ε_e_	0.277 (0.128–0.431) *	
TLAEF	7.040 (2.403–11.676) *		ALAEF	12.599 (1.748–23.450) *	

Note: Abbreviation of ε_e_, GLPS, LVEF, TLAEF and ALTAEF are showing in Table 2

* P less than 0.05

### Reproducibility

The intraobserver and interobserver reproducibility of LA strain and strain rate were considered excellent (all ICCs > 0.8). The intraobserver and interobserver correlation coefficients are shown in the supplementary material (table S1).

## Discussion

This study focused on the atrioventricular interaction effects in T2DM patients with or without anemia. The main findings were as follows: [1] Compared with controls and T2DM patients without anemia, T2DM patients with anemia had higher LA phasic volumes and severely impaired LAEF, LA phasic strain and LV GLPS [2]. Hb, HbA1c and LV GLPS were independent factors influencing LA phasic strains [3]. LA phasic function and LV function interacted with each other.

### Characteristics of T2DM patients with anemia

In this study, T2DM patients with anemia were the oldest and had the highest heart rate, highest creatinine, lowest eGFR and lowest Hb. When patients suffer from anemia, the body compensates to remedy the insufficient oxygen supply, such as by reducing afterload, increasing preload, and increasing positive inotropic and chronotropic effects [7]. One of these manifestations occurring in the heart is increased heart rate [22]. In addition, the fact that older people are more likely to develop anemia is consistent with our findings. Many reasons, such as chronic inflammatory diseases, nonhematopoietic neoplasms, endocrinologic and metabolic causes, blood loss, and lack of nutrients, lead older people to be more likely to develop anemia [23]. Diabetic kidney disease may affect ∼50% of T2DM patients and can remarkably worsen the prognosis of T2DM patients [24, 25]. In our study, patients with anemia had lower eGFR and higher creatinine, which meant that their renal function was impaired to varying degrees. Once renal function decreases, the production of erythropoietin declines, and then the production of red blood cells also decreases (which is called renal anemia) because the kidney is the most important organ for erythropoietin production in adults [26]. T2DM is also more likely to lead to anemia even when kidney function is normal [27]. Whether anemia is combined with T2DM, chronic kidney disease or both, anemia is associated with lower quality of life and higher mortality [28, 29]. Thus, when patients have T2DM, anemia should be assessed to optimize the prognosis.

### Controversial LA phasic volumetric and LV volumetric functions of T2DM patients with anemia

In our study, almost all LA volumetric functions were impaired in the T2DM patients with anemia. Some LV volumetric functions were impaired, while others were relatively preserved. When patients have anemia, the oxygen carrying capacity of blood is reduced. In response to hypoxia, one of the compensatory mechanisms is to transfer more blood to tissue. As stated above, increased preload and increased cardiac output are among the body’s compensation mechanisms [7, 22]. Thus, volume overload caused by anemia might be one of the reasons that the LAVI_pre−a_, LAVI_min_, LVESV and LVEDV of T2DM patients with anemia increased to varying degrees in our study. However, when patients have T2DM, cardiac function is damaged by cardiac insulin resistance and hyperglycemia [30, 31]. These factors result in increased LVM, reduced LVEF and even heart failure [32]. In our study, we also observed an increase in LVM and a decrease in LVEF in T2DM patients. Anemia would lead to a volume increase, but anemia itself and the remedy for it can also damage the heart. In the condition of anemia, the heart oxygen supply might be insufficient. Increased positive inotropic and chronotropic effects might not only increase heart workload but also reduce the relaxation time of the heart, which could further damage the oxygen supply [22]. The combined impacts of anemia and T2DM help to explain our results that T2DM patients with anemia had some controversial left heart volume parameters and worse LA and LV strain, that is, the highest LAVI, the worst LA and LV global longitudinal strains, slightly higher LVEDVI and LVESVI, but relatively normal LVSV, CO and CI.

### The left atrioventricular interaction in all T2DM patients

By univariate and multivariate linear regression analyses, LV GLPS had the greatest independent influence on the LA phasic strain of T2DM patients, while ε_e_, total LAEF and active LAEF were independent factors of LV function. LA function can be divided into three phases: LA reservoir function, LA conduit function and LA pump function. On the one hand, LA phasic function heavily relies on LV performance. Before mitral valve opening, the LA stores blood from the pulmonary circulation, and its reservoir function is regulated by LV contraction, pulmonary circulation pressure and the nature of the LA. During early left ventricular diastole, the LA transfers blood from the pulmonary circulation to the left ventricle, and its conduit function is mainly regulated by LV diastolic properties. During late left ventricular diastole, the LA pumps blood to the left ventricle and is regulated by the nature of LA and LV compliance and LV end-diastolic pressure [33, 34]. On the other hand, LA can regulate LV filling pressure and cardiac output [35]. LA phasic function is strongly associated with LV diastolic dysfunction and could be conducive to LV systolic dysfunction [34, 36]. The left atrium and left ventricle maintain a close dynamic interaction during the cardiac cycle. Once one part of them is impaired, another will also suffer, and a vicious circle might consequently develop. Reduced LV function leads to poor quality of life and high mortality [37], and impaired LA function is also associated with poor prognosis in many cardiovascular diseases [15]. Fortunately, timely treatment can lead to LA and LV reverse remodeling, which includes functional and structural reverse remodeling [38, 39]. As shown in this study, both LA and LV function are severely impaired in T2DM patients with anemia. Thus, it is important to timely supervise and improve the LA and LV function of T2DM patients with anemia concurrently to improve their quality life and their prognosis.

### Limitations

Some limitations of this study should be mentioned. First, potential selection bias might be unavoidable due to the single-center and retrospective nature of this study. Second, we only used global strain to explore the relationship between LV strain and LA strain because global strain has a higher reproducibility than regional strain parameters [40]. Finally, echocardiography can also be used to examine LA and LV function, but our results did not include echocardiography results for LA, and LV strain was not routinely examined by echocardiography. In the future, we would make up for this limitation by enrolling more patients who undergo relative examination.

## Conclusion

Anemia and T2DM were independently associated with LA phasic strain function, and LA phasic function was severely impaired in T2DM patients with anemia. Adverse interaction between LA phasic function and LV function may happen in T2DM patients with or without anemia. To improve T2DM patients’ quality of life and prognosis, timely monitoring and treatment of both LA function and LV function might be indispensable.

## Electronic supplementary material

Below is the link to the electronic supplementary material.

Supplementary Material 1

## Data Availability

The datasets generated during and/or analyzed in the current study are available from the corresponding author upon reasonable request.

## Abbreviations

CMR: Cardiac magnetic resonance
DBP: Diastolic blood pressure
eGFR: Estimated glomerular filtration rate
GLPS: Global longitudinal peak strain
HDL: High-density lipoprotein
Hb: Hemoglobin
ICCs: Intraclass correlation coefficients
LA: Left atrial
LAEF: Left atrial emptying fraction
LAV_max_: The maximum LA volume
LAV_min_: The minimum LA volume
LAV_pre−a_: The preatrial contraction LA volume
LV: Left ventricular
LDL: Low-density lipoprotein
LVEDVI: Left ventricular end-diastolic volume index
LVEF: Left ventricular ejection fraction
LVESV: Left ventricular end-systolic volume
LVM: Left ventricular mass
LVSV: Left ventricular stroke volume
SBP: Systolic blood pressure
T2DM: Type 2 diabetes mellitus
TC: Total cholesterol
TG: Triglyceride
ε_a_: Left atrial active strain
ε_e_: Left atrial passive strain
ε_s_: Left atrial reservoir strain
