# Supplementary material for: Left atrioventricular interaction and impaired left atrial phasic function in type 2 diabetes mellitus patients with or without anemia: a cardiac magnetic resonance study
Source: Cardiovasc Diabetol. 2023 Jul 13;22:178. doi: 10.1186/s12933-023-01910-8 (PMC10347718; doi:10.1186/s12933-023-01910-8)
Supplement: Supplementary file 1 — Supplementary Material 1 [file 12933_2023_1910_MOESM1_ESM.docx]

table S1 Intraobserver and interobserver variabilities

|  | **Intra-observer** | | **Inter-observer** | |
| --- | --- | --- | --- | --- |
|  | ICC | 95%CI | ICC | 95%CI |
| ε_s_ | 0.962 | 0.925 – 0.981 | 0.918 | 0.843 – 0.958 |
| ε_e_ | 0.927 | 0.858 – 0.963 | 0.892 | 0.796 – 0.945 |
| ε_a_ | 0.935 | 0.874 – 0.967 | 0.818 | 0.666 – 0.905 |

Note: Abbreviation of ε_s_, ε_e_ and ε_a_ are shown in Table 2
